# Comparison of pre-and postoperative medication costs in patients who underwent bariatric surgery – a nationwide data analysis

**DOI:** 10.3389/fpubh.2024.1344040

**Published:** 2024-02-08

**Authors:** Magdalena Osińska, Yaroslav Sanchak, Andrzej Śliwczyński, Edward Franek, Magdalena Walicka

**Affiliations:** ^1^Department of Internal Diseases, Endocrinology and Diabetology, National Medical Institute of the Ministry of the Interior and Administration, Warsaw, Poland; ^2^National Medical Institute of the Ministry of the Interior and Administration, Warsaw, Poland; ^3^Department of Human Epigenetics, Mossakowski Medical Research Institute, Polish Academy of Sciences, Warsaw, Poland

**Keywords:** obesity, bariatric surgery, cost-effectiveness, cost, overweight

## Abstract

**Introduction:**

Bariatric surgery has known health benefits and may lower the medication-related costs. This study aimed to assess the cost of medications prior to and after bariatric surgery in the Polish nationwide registry.

**Methods:**

The study included 2,390 adults. The analysis was conducted separately for a 12-month pre-operative period, and a 12-month postoperative period. The total costs of medication and cost per anatomical therapeutic chemical group were assessed and the mean cost per patient in the preoperative and postoperative periods was compared.

**Results:**

The study showed a significant increase in the overall medication costs and mean costs of medications per patient in the year after bariatric surgery. This increase was related mainly to low-molecular-weight heparins used in the 1st month after surgery. Alternatively, costs of medication used in the cardiovascular system diseases and anti-infectives decreased significantly. The total costs of hypoglycemic agents were reduced by 46%, antihypertensive medications by 29%, and lipid-lowering drugs by 38.

**Conclusions:**

In general, medication costs are higher in the first year after surgery. The increase results from the perioperative use of low-molecular-weight heparins, whereas a significant cost reduction of glucose-, lipid-lowering, antihypertensive, and anti-infective medications was observed.

## 1 Introduction

According to estimates by the World Obesity Federation, the prevalence of overweight and obesity (BMI ≥25 kg/m^2^) will increase from 38% of the world's population in 2020 to over 50% in 2035. That being so, by 2035 over 4 billion people (over 5 years of age) will be living with these conditions (1). In the National Study of Nutrition and Nutritional Status of the Polish Population published in 2021, overweight was found in 52.4% of men and 32% of women, and obesity in 16.5% and 16.2%, respectively (2). Accordingly, overweight and obesity are becoming a pandemic and a significant public health problem all over the world.

Obesity is a disease that involves multiple organ systems which increases the risk of the most common non-communicable chronic diseases (type 2 diabetes, hypertension, cardiovascular diseases, non-alcoholic fatty liver disease, obstructive sleep apnea, hypoventilation syndrome, osteoarthritis, cancer, mood, and anxiety disorders) (3). Thus, it is not surprising that obesity treatment costs are high. In 2019, per capita costs of obesity ranged from USD 17 in India to USD 940 in Australia (4). Obesity raises the costs of inpatient, and outpatient care, as well as prescription medication. In the US, the effects of obesity increase the ambulatory care expenditures by 67.0%, prescription drug expenditures by 186.8% (excluding anti-obesity medications), and inpatient services by 177.6% and 923.9% for class 1 and class 3 obesity, respectively (5).

Lifestyle modification (diet and physical activity) is a necessity for successful, long-term weight reduction. Bariatric surgery is however the most effective tool in the treatment of obesity. It is associated with substantial and durable weight loss (6). It is an effective procedure not only in the treatment of obesity but also in the prevention and control of comorbidities (7, 8). This improvement should be linked to the reduction of the number of medications taken and their related costs. On the other hand, bariatric surgery is an invasive procedure that can be associated with complications such as a stenosis, anastomotic leak, postoperative bleeding, venous thromboembolism, infections, megaesophagus or pseudoachalasia, gastroesophageal reflux disease, ulcers, several nutritional deficits, internal hernias, gallstone disease, and dumping syndrome (9–11) which may require additional procedures and medications, resulting in an increase of the cost of care. It is well known that patients who have undergone obesity surgery require supplementation of multivitamins and minerals (12). In most bariatric centers, proton pump inhibitors are also prescribed to minimize the risk of ulcer formation and gastroesophageal reflux symptoms. Taking all this into account, lowering medication costs after bariatric surgery is not obvious. Previous studies have demonstrated a decrease in medication costs after surgery (13) as well, showing similar costs for surgically treated obese individuals when compared to those treated conventionally (14). In our previous study assessing the cost of surgical treatment of obesity and its impact on healthcare expenses, the total costs of medications decreased 1 year after obesity surgery, there was however some disparity between the medication costs per patient expressed as a median and mean (15). Thus, the study performed a detailed analysis of the changes in drug costs during the twelfth-month periods before and after bariatric surgery in the Polish nationwide registry.

## 2 Materials and methods

A retrospective design was used to analyze the national database. The study included adult patients having undergone bariatric surgery during the period of January 1, 2017, to September 30, 2017 (2017 was selected for analysis because during this year the Agency for Health Technology Assessment and Tariff System in Poland separated bariatric surgery from general surgery and created a new group of guaranteed services called surgical treatment of obesity, reimbursed by the National Health Fund). This sample of patients undergoing bariatric surgery was acquired from the National Health Fund (NHF) database. NHF is the state-owned financer of all reimbursed healthcare procedures across the country which requires that healthcare providers who had signed an agreement with this organization regarding the provision of healthcare services are required to maintain an electronic patient registry with the NHF. The data included within this registry includes, among others, information such as the patient's personal identifying number, the International Statistical Classification of Disease and Related Health Problems (ICD-10) disease code, and the Diagnosis Related Group for a given service. With regards to bariatric surgery, the Diagnosis Related Group was classified as F17. In a similar way, pharmacies are required to maintain and provide a record of filled prescriptions to the NHF. The provided record also includes, among others, the patients personal identifying number and the code number (European Article Number EAN) of medication. For this study, the sample was generated by taking all services provided for the Diagnosis Related Group F17 and the associated filled prescriptions for the associated personal identifying numbers. The prescribed medications were then categorized in accordance with the Anatomical Therapeutic Chemical Classification System (ATC). The total cost of medications and the cost of each individual ATC group were assessed. An analysis was carried out separately for prescriptions used in the treatment of diabetes, hypertension, and hyperlipidemia. It was decided to perform a separate analysis of these prescriptions, because it is well known that obese compared to non-obese individuals use more cardiovascular and diabetes medications (16) and cost changes of these medications after surgery seem particularly important. The mean cost, per personal identifying number, within the preoperative period (a period of 12 months before the surgery), and the postoperative period (a period of 12 months after the surgery) were compared. The median of the differences between costs of the two periods was calculated.

### 2.1 Statistical analysis

Statistical analysis was performed using the R Project for Statistical Computing program (version 3.5.1.). Preoperative and postoperative costs were presented as absolute and percent values. Data for costs for the comparison of pre- and postoperative periods were presented using measures of central tendency (mean and median) and dispersion (standard deviation and interquartile range). The normality of distribution was tested using the Kolmogorov–Smirnov test as well as based on data skewness and kurtosis values. A comparison of preoperative and postoperative costs was performed using the non-parametric Wilcoxon test for paired samples because the assumptions of the parametric test were not met (high skewness and kurtosis of the data, lack of normality of the distribution). All tests performed were two-sided with a significance level of 0.05.

## 3 Results

The study included 2,390 patients (601 men and 1,789 women). With reference to preoperative costs, the total cost for the treatment of the entire group of patients in the year preceding surgery amounted to PLN 6.91 million (USD 1.73 million). The cost attributed to medications accounted for PLN 1.03 million (USD 0.3 million, 15% of total costs). The highest cost was incurred for medications used to treat diseases of the alimentary tract and metabolism (group A according to the ATC classification) - PLN 262, 010 (USD 65,503), followed by drugs used in diseases of the cardiovascular system (group C) - PLN 238,989 (USD 59,747). Next was the cost of drugs used in diseases of the respiratory system (group R) - PLN 106,748 (USD 26,696) and the nervous system (group N) - PLN 103,824 (USD 25,956).

With reference to the postoperative period, the total treatment cost for the entire group of patients in the year post surgery totaled to PLN 31.4 million (USD 7.85 million) and most of it was related to the cost of the bariatric surgery itself (PLN 26.29 million, USD 6.57 million). The cost attributed to medications accounted for almost PLN 1.3 million (USD 0.33 million, 4% of total cost). The highest cost was incurred for medications used in the treatment of blood and blood forming organs (group B) - PLN 199,388 (USD 49,847), followed by drugs used in the diseases of alimentary tract and metabolism (group A) – PLN 199,388 (USD 49,847). Next was the cost of drugs used in diseases of the cardiovascular system (group C) - PLN 168,488 (USD 42,122) and the nervous system (group N) - PLN 135,184 (USD 33,796). Costs of pharmacotherapy in the first 30 days after surgery accounted for PLN 302,945 (USD 75,736) and were generated mainly by low-molecular-weight heparins (PLN 240,645, USD 60,161) and proton pump inhibitors (PLN 42,668, USD 10,667).

Detailed preoperative and postoperative costs of individual groups of drugs (according to ATC classification) are presented in Table 1.

**Table 1:** Preoperative and postoperative yearly costs of individual groups of drugs (according to ATC classification) in Poland, according to NHS registry.

**ATC category**	**Description**	**Preoperative cost**		**Postoperative cost**	
		**Value, PLN**	**% of total medication cost**	**Value, PLN**	**% of total medication cost**
A	Alimentary tract and metabolism	262,010.22	25.4	199,388.39	15.7
B	Blood and blood forming organs	49,512.33	4.8	329,135.80	25.9
C	Cardiovascular system	238,988.66	23.1	168,487.81	13.3
D	Dermatological	3656.05	0.4	5330.23	0.4
G	Genito-urinary system and sex hormones	17,273.55		19,087.46	1.5
H	Systemic hormonal preparations, excluding sex hormones, and insulins	16,972.99	1.7	20,483.70	1.6
J	Anti-infectives for systemic use	79,144.27	7.7	55,317.28	4.4
L	Antineoplastic and immunomodulating agents	10,830.72	1.0	45,784.07	3.6
M	Musculo-skeletal system	36,682.41	3.5	36,307.23	0.3
N	Nervous system	103,823.71	10.0	135,184.08	10.6
P	Antiparasitic products, insecticides, and repellents	462.43	0.04	563.53	0.04
R	Respiratory system	106,748.14	10.3	83,433.66	6.6
S	Sensory organs	2572.99	0.2	3557.62	0.3
V	Various	66,456.80	6.4	69,302.48	5.5
	No drug code	38,268.54	3.7	99,153.73	7.8
	Total medications' cost	1,033,403.81	100	1,270,517.07	100

Percentages do not add up to 100% due to rounding of numbers to one decimal place. To convert the values in PLN to USD they should be divided by 4 (USD 1 = PLN 4).

Analysis of the mean cost per patient revealed that the total cost of the medications 12 months after surgery was significantly higher than before. This increase was generated mainly by the medications used to treat blood and blood-forming organs (group B). In this group, the main cost was related to low-molecular-weight heparins. A significant increase in cost was also observed regarding medicines used in the treatment of alimentary tract and metabolism diseases (group A). A significant decrease was concerned with the cost of medications used in cardiovascular system diseases (group C), and anti-infectives for systemic use (group J). Average preoperative and postoperative cost per patient for each medications group (according to ATC classification) were presented in Table 2.

**Table 2:** Average preoperative and postoperative cost of each medication group according to ATC classification (per capita in PLN yearly).

**ATC category**	**Description**	**Preoperative cost**		**Postoperative cost**		***P*-value**
		**Me (IQR)**	**Mean** ±**SD**	**Me (IQR)**	**Mean** ±**SD**	
A	Alimentary tract and metabolism	19.29 (75.64)	110.65 ± 367.19	33.99 (74.45)	84.20 ± 223.60	< 0.001
B	Blood and blood forming organs	0.00 (0.00)	20.91 ± 120.54	112.62 (155.98)	138.99 ± 283.33	< 0.001
C	Cardiovascular system	0.00 (143.65)	100.92 ± 169.13	0.00 (68.65)	71.15 ± 150.47	< 0.001
D	Dermatological	0.00 (0.00)	1.54 ± 18.81	0.00 (0.00)	2.25 ± 27.35	0.081
G	Genito-urinary system and sex hormones	0.00 (0.00)	7.29 ± 48.51	0.00 (0.00)	8.06 ± 59.14	0.428
H	Systemic hormonal preparations, excluding sex hormones, and insulins	0.00 (0.00)	7.17 ± 20.47	0.00 (0.00)	8.65 ± 24.76	0.737
J	Anti-infectives for systemic use	17.76 (49.32)	33.42 ± 55.03	0.00 (31.12)	23.36 ± 111.80	< 0.001
L	Antineoplastic and immunomodulating agents	0.00 (0.00)	4.57 ± 93.94	0.00 (0.00)	19.33 ± 439.03	0.864
M	Musculo-skeletal system	0.00 (15.80)	15.49 ± 34.70	0.00 (15.79)	15.33 ± 36.16	0.506
N	Nervous system	0.00 (0.00)	43.84 ± 293.25	0.00 (0.00)	57.09 ± 441.59	0.101
P	Antiparasitic products, insecticides, and repellents	0.00 (0.00)	0.20 ± 3.18	0.00 (0.00)	0.24 ± 5.32	0.712
R	Respiratory system	0.00 (0.00)	45.08 ± 196.14	0.00 (0.00)	35.23 ± 186.06	0.013
S	Sensory organs	0.00 (0.00)	1.09 ± 17/04	0.00 (0.00)	1.50 ± 22.76	0.273
V	Various	0.00 (0.00)	28.06 ± 117.77	0.00 (0.00)	29.27 ± 131.90	0.773
	No drug code	0.00 (0.00)	16.16 ± 79.72	0.00 (0.00)	41.87 ± 110.92	< 0.001
	Total medications' cost	207.74 (443.8)	436.40 ± 713.03	318.77 (409.6)	536.54 ± 923.04	< 0.001

SD, standard deviation; Me, median; IQR, interquartile range; p-value in Wilcoxon test. To convert the values in PLN to USD they should be divided by 4 (USD 1 = PLN 4).

With reference to the postoperative period, the total costs of hypoglycemic agents were reduced by 46%, antihypertensive medications by 29%, and lipid-lowering drugs by 38%. The total cost of glucometer strips has not changed. Average preoperative and postoperative costs per patient were presented in Table 3.

**Table 3:** Average preoperative and postoperative costs of medications used in the treatment of diabetes, hypertension and hyperlipidemia (per capita in PLN yearly).

**Medications**	**Preoperative cost**		**Postoperative cost**		***P*-value**
	**Me (IQR)**	**Mean** ±**SD**	**Me (IQR)**	**Mean** ±**SD**	
Antidiabetic medications (all)	0.00 (18.84)	109.58 ± 429.21	0.00 (0.00)	58.63 ± 282.88	< 0.001
Metformin	0.00 (4.91)	25.84 ± 61.06	0.00 (0.00)	11.39 ± 43.23	< 0.001
Insulin	0.00 (0.00)	50.59 ± 325.80	0.00 (0.00)	18.01 ± 188.21	< 0.001
Other antidiabetic medications	0.00 (0.00)	33.15 ± 126.35	0.00 (0.00)	29.23 ± 129.50	< 0.001
Antihypertensive medications	0.00 (90.14)	66.30 ± 120.24	0.00 (29.68)	46.75 ± 108.16	< 0.001
Lipid-lowering medications	0.00 (0.00)	19.43 ± 58.02	0.00 (0.00)	12.03 ± 45.60	< 0.001

SD, standard deviation; Me, median; IQR, interquartile range; p-value in Wilcoxon test. To convert the values in PLN to USD they should be divided by 4 (USD 1 = PLN 4).

## 4 Discussion

Our study revealed a significant overall increase in the medication cost 1 year after bariatric surgery. This result contradicts those of Nguyen et al. (13) who showed that the mean monthly medication savings for the first year after surgery was USD 168, with a yearly savings of USD 2016 per patient. Similarly, Keating et al. (17) observed that when compared to the 12 months before bariatric surgery, overall pharmaceutical utilization fell in the 24 months after the procedure and costs decreased from AUD 517 to AUD 435 per person.

The increase in the medication cost in our study was largely due to the cost of low-molecular-weight heparin in the first month after surgery. Likewise, in the study by Gesquiere et al. (18) a 36-fold increase in the cost of medications 1 month after surgery (compared to the period before surgery) was observed, which was caused by, among others, the use of low-molecular-weight heparin (LMWH). This is not surprising because thromboprophylaxis in people undergoing bariatric surgery is very important. Venous thromboembolism (VTE) in this group of patients is associated with readmission and significantly increased mortality (19). One of the major problems with thromboprophylaxis in bariatric surgery is however the poor knowledge about the effectiveness of thromboprophylaxis regimens in the obese population. Too little high-quality research, as well as the doubts about the appropriate doses of LMWH in obese patients make it difficult to determine the optimal recommendations. Some studies point to extended outpatient thromboprophylaxis in patients after obesity surgery because most VTE events occur after hospital discharge (20), however according to the recent meta-analysis, current evidence is insufficient to support this proposal (21). Recommendations developed by the American Association of Clinical Endocrinologists (AACE), The Obesity Society (TOS), the American Society for Metabolic and Bariatric Surgery (ASMBS), the Obesity Medicine Association (OMA), and the American Society of Anesthesiologists (ASA) suggest thromboprophylaxis (subcutaneously administered unfractionated heparin or low-molecular-weight heparin), given within 24 hours after operation for all patients after bariatric surgery (22). In turn, the recommendations of enhanced recovery after surgery (ERAS) society, updated in 2021, do not specify the scheme of prophylaxis. Its authors only recommend individualized doses and duration of treatment (23). Accordingly, the Polish recommendations in the field of bariatric and metabolic surgery, thromboprophylaxis should include low-molecular-weight heparin, the dose of which should be selected individually. Continuation of thromboprophylaxis (preferably with the use of LMWH) is valid for at least 7 days after the procedure (24). The use of different prophylaxis regimens may account for differences in postoperative drug costs between countries.

Interestingly we found a significant decrease in the costs of anti-infectives for systemic use in the postoperative period. This may indicate a lower incidence of infections after bariatric surgery. The effect of obesity surgery and weight loss on infectious diseases risk in people with obesity has not been fully studied. Goto et al. (25) showed decreased risk of skin and soft tissue infection and respiratory infections, and increased risk of intra-abdominal infection and urinary tract infection after weight loss surgery. In the study performed by Cundy et al. (26) hospitalization for skin and soft tissue, urinary tract, and lower respiratory tract infections in people after bariatric surgery was lower than in those enrolled in the bariatric program but did not undergo operation. Likewise, the results of a study by Valera et al. (27) suggest that the risk of hospitalization due to influenza may fall after bariatric surgery. In turn, in relation to meta-analysis of observational studies it seems that obesity surgery is related to less severe COVID-19 (28). To assess the influence of bariatric surgery on the risk of infectious diseases, additional, randomized controlled trials are needed.

Our study showed that in the postoperative period, the total costs of hypoglycemic agents were reduced by 46% (the largest decrease was observed in insulin cost). Similarly, in the study by Gesquiere et al. (18), the costs of prescriptions for medications used in the treatment of type 2 diabetes 1 year after obesity surgery was reduced by 85%. Likewise, in the study of Swedish Obese Subjects (SOS) (14) the costs of medication used in diabetes treatment decreased significantly after bariatric surgery. In turn, Wu et al. (29) demonstrated that for patients who had undergone bariatric surgery, the costs of diabetes drugs illustrate a U-shape curve. The costs of medication in patients after operation were shown to drop 2 years after surgery (€1752 in year 1 and €973 in year 2) and rise by year 5 (to €1836). The authors point out; however, the reduction in the number of insulin prescriptions.

Despite a significant reduction in the costs of antidiabetic drugs, we did not show a change in spendings on glucose meter test strips. The explanation for this result is probably frequent glycemic control carried by patients due to the changing demand for glucose-lowering medications. All of these results are not surprising because many studies have shown significant improvement of glucose control after bariatric surgery and even diabetes remission (30, 31). According to the review performed by Affinati et al. (32) diabetes remission is observed in 33%−90% of individuals 1-year post obesity surgery. It should be noted that in people after bariatric surgery, the highest incidence of discontinuation and the lowest incidence of restart is found in diabetes medications when compared with antihypertensive and lipid-lowering medications (33).

Likewise, regarding the costs of antihypertensive and lipid-lowering medications, we observed significant changes in the postoperative period. The cost of antihypertensive drugs dropped by 29%, and lipid-lowering by 38%. In the study by Ghiassi et al. (34), the annual cost of anti-hypertensives fell by 65%. Likewise, Morton et al. (35) showed significant reduction of the cost related to hypertension treatment in the first year after surgery. The observed downturn of cost is most likely the result of the improvement of blood pressure control and correction of lipid disorders, and even remission of these cardiovascular risk factors the after bariatric surgery. This is indicated by many research results. In the meta-analysis performed by Chang et al. (36) remission rate of hypertension was 75% for randomized controlled trials and 74% for observational studies, and dyslipidemia respectively 76% and 68%.

Our investigation has some limitations. The first one is its retrospective nature. As a result, it was not possible to obtain, for a given patient, information on obesity categories, and comorbidities as well as on the type of operation performed. Additionally, we could not obtain data regarding the other methods of treating obesity, thereby making the comparison of the costs of the medications between patients treated non-surgically and surgically was impossible. The follow-up period was also short, mainly because of the COVID pandemic, resulting in a significantly reduced number of elective surgeries and ambulatory visits to physicians. The relatively short duration of the study and additionally, the lack of a control group restrained the study's ability to conclude how obesity surgery influences the expenses of medication. It should be taken into consideration, however, that the National Health Fund database of Poland is the largest healthcare database of this country, which all performed public healthcare services are registered. Therefore, an assumption can be made that the costs reported in this study are direct representatives of the country's spending on medications in patients treated with weight loss surgery. Additionally, this study benefits from a high reliability of the conclusions, given the large group of patients observed.

## 5 Conclusion

This analysis shows that medication costs in patients who underwent bariatric surgery are higher in the first year after bariatric surgery compared to the preoperative period. This increase in costs is mainly influenced by the cost of low-molecular-weight heparins. There is a significant cost reduction of glucose-, lipid-lowering, antihypertensive, and anti-infective medications in the postoperative period. Further studies with a longer observation period, a control group and cost-effectiveness modeling are needed to further clarify how bariatric surgery influences medication costs.

## Data availability statement

The original contributions presented in the study are included in the article/supplementary material, further inquiries can be directed to the corresponding author.

## Ethics statement

Ethical review and approval were waived for this study due to the retrospective and non-invasive nature of the study (retrospective analysis of nationwide database). The studies were conducted in accordance with the local legislation and institutional requirements. Written informed consent for participation was not required from the participants or the participants' legal guardians/next of kin in accordance with the national legislation and institutional requirements because informed consent from patients is not required due to the retrospective and non-invasive nature of the study.

## Author contributions

MO: Investigation, Writing—original draft, Writing—review & editing. YS: Writing—original draft, Writing—review & editing, Visualization. AŚ: Conceptualization, Data curation, Resources, Writing—original draft, Validation. EF: Conceptualization, Methodology, Validation, Supervision, Writing—review & editing. MW: Conceptualization, Supervision, Validation, Writing—original draft, Writing—review & editing, Visualization.
